# Midterm postoperative prognosis of patients with severe left heart valvular disease combined with moderate or severe pulmonary hypertension treated with treprostinil

**DOI:** 10.1186/s12872-020-01759-9

**Published:** 2020-11-03

**Authors:** Ning Xu, Shu-Ting Huang, Kai-Peng Sun, Zeng-Chun Wang, Hua Cao, Qiang Chen

**Affiliations:** 1 Department of Cardiac Surgery, Fujian Maternity and Child Health Hospital, Affiliated Hospital of Fujian Medical University, Fuzhou, China; 2 Department of Cardiovascular Surgery, Union Hospital, Fujian Medical University, Fuzhou, China

**Keywords:** Treprostinil, Postoperative effect, Valvular disease, PAH

## Abstract

**Background:**

To investigate the midterm postoperative prognosis of patients with severe left heart valvular disease combined with moderate or severe pulmonary hypertension (PAH) using subcutaneous injection of treprostinil.

**Methods:**

A retrospective study was conducted on 61 patients with severe left heart valvular disease combined with moderate or severe PAH who had undergone mechanical mitral and/or aortic valve replacement from April 2018 to October 2018. The patients were divided into the treprostinil group and the conventional treatment group according to whether they received treprostinil. The patients were assessed by SwanGanz catheterization, echocardiography, the 6-min walk test (6-MWT), the Borg dyspnoea score and the SF-36 questionnaire.

**Results:**

Compared with the preoperative data, the mPAP measured by SwanGanz catheterization, the results of the 6-MWT and the Borg score were significantly improved in both groups during the 1 year follow-up (P < 0.05). Regarding the comparison between the groups, the results in group T were significantly better than those in group C, including the results of the 6-MWT and the general health, vitality and mental health of SF-36 during the 1 year follow-up (P < 0.05).

**Conclusions:**

Continuous subcutaneous infusion of treprostinil was not capable of decreasing pulmonary pressures in patients with severe left heart valvular disease combined with moderate or severe PAH during 1 year follow-up, although which some of our data suggest that might improve the symptoms and quality of life of these patients.

## Background

Studies have shown that pulmonary hypertension (PAH) is found in almost all patients with severe mitral stenosis and half of patients with symptomatic aortic stenosis, and the prevalence of PAH in patients with left heart valvular disease increases with the severity of valvular disease and aggravation of symptoms [1]. Prosthetic valve replacement remains the best choice for the treatment of left heart valve disease [2]. However, the ability to use prosthetic valve replacement to completely address PAH is a misconception. Many clinical studies have confirmed that the persistence of PAH after valve replacement is common, and PAH remains an important factor leading to a poor postoperative prognosis [3–5]. In clinical practice, the patients’ symptoms and quality of life may not improve significantly after surgery due to the presence of PAH, not only affecting the postoperative recovery of patients but also leading to the worsening of the disease [6, 7]. In addition to routine treatment with cardiotonic drugs, diuresis and vasodilators, the use of targeted drugs is also gradually increasing. As a stable prostacyclin analogue, treprostinil plays an important role in reducing pulmonary artery pressure [8–10]. Based on a literature search, few studies exist on the use of treprostinil in patients with PAH caused by left heart valve disease. Therefore, this retrospective study mainly discussed the midterm postoperative prognosis of patients with severe left heart valvular disease combined with PAH who were treated with subcutaneous injection of treprostinil.

## Methods

This was a retrospective study. According to the pre-survey results, the alpha value was set to 0.05, and the power was set to 0.90. The resulting minimum sample size was 56 patients in this study based on the calculation. So the clinical data of 61 patients with severe left heart valvular disease combined with PAH who had undergone mechanical mitral and/or aortic valve replacement at our hospital from April 2018 to October 2018 were retrospectively analyzed in this study.

All the patients were diagnosed with mitral and/or aortic valve lesions with or without tricuspid valve lesions by transthoracic echocardiography (TTE). Moderate or severe PAH was also diagnosed in these patients based on the following criteria: pulmonary artery systolic pressure (PASP) > 50 mmHg estimated by TTE and the mean pulmonary artery pressure (mPAP) > 35 mmHg measured by SwanGanz catheterization [11]. The age of the patients was older than 18 years, and the doctor’s advice was followed. The exclusion criteria were as follows: 1. the presence of other types of PAH, such as chronic thromboembolic PAH, hypoxia or pulmonary disease-associated PAH, 2. repeat heart surgery, and 3. severe liver or kidney failure, malignant tumour or severe somatic dysfunction.

During the preoperative hospitalization period, the patients performed a 6-min walk test (6-MWT) without assistance and scored their level of exertion on the Borg scale. After obtaining consent from the patients, the patients completed the SF-36 questionnaire and were informed of the questionnaire requirements and precautions in the standard language. Illiterate and semi-illiterate patients completed the questionnaire with guidance from their families.

After routine preoperative treatment, all the patients received surgical treatment and achieved successful surgery. Based on the patient’s physical condition, financial situation, perioperative treprostinil use, and the patient’s wishes, all the patients were divided into the treprostinil group (group T, N = 21) and conventional treatment group (group C, N = 40) according to whether they received treprostinil after discharge. The basic information of the patients is listed in Table 1. Preoperative consultation with patients and their families about subsequent anti-pulmonary hypertension drug treatment was conducted. Patients in group C only received vasodilators, cardiac drugs and diuretics, and patients in group T received treprostinil infusion via a positive pressure microinjection pump in addition to those treatments described above. The infusion tube was placed in subcutaneous tissues of the abdomen. Starting from 1.25 ng/(kg·min), the dosage was adjusted according to the disease condition and patient tolerance (maximum dose 20 ng/(kg·min)), and the dose was continued to be used 12 months after discharge. The subcutaneous pump was checked regularly to ensure proper function.

**Table 1:** General clinical information in both groups

Items	Group T (n = 21)	Group C (n = 40)	*P*-value
Age (year)	58.0 [53.0, 61.0]	59.0 [53.0, 64.3]	0.252
Gender (female/male)	13/8	25/15	0.964
Valvular surgery (single valve replacement/double valve replacement)	18/3	36/4	0.618
PASP (mmHg)	67.0 [63.0, 74.0]	65.5 [62.5, 71.3]	0.261
Ejection fraction (%)	61.3 [54.8, 66.3]	62.3 [55.4, 68.7]	0.872
LVEDD (mm)	56.3 [53.8, 59.1]	56.1 [49.1, 61.6]	0.723
HR (times)	81.0 [75.0, 87.0]	82.0 [76.0, 89.0]	0.542
mPAP (mmHg)	42.0 [38.0, 47.0]	43.0 [40.0, 46.3]	0.979
NYHA	4/13/4	6/27/7	0.895
6-MWD (m)	352 [312, 376]	336 [284, 375]	0.832
Borg score	5.00 [4.00, 5.00]	5.00 [4.00, 5.00]	0.891
CPB time (min)	82.0 [77.0, 115]	88.5 [78.8, 116]	0.802
Aortic cross-clamp time (min)	44.0 [37.0, 69.0]	44.5 [40.0, 69.3]	0.811
Surgery time	123 [101, 145]	133 [122, 145]	0.529

The patients were returned to our hospital for the assessment at 3 months and 1 year after discharge; otherwise, they visited any time if they felt unwell. The 6-MWT and Borg dyspnoea index were performed at each visit. TTE was also performed 3 months after surgery; additionally, SwanGanz catheterization was examined, and the SF-36 questionnaire was administered to evaluate the quality of life 1 year after the surgery.

The patients were told to try their best to walk back and forth for 6 min down a 30-m corridor, and the 6-min walking distance (6-MWD) and discomfort response during walking were recorded. The heart rate, blood pressure and pulse oxygen were measured before and after the test. The Borg dyspnoea score was assessed at the end of the 6-MWT. This score reflected the maximum breathing difficulty the patients experienced at any time during the 6-MWT. When the walking distance was longer and Borg’s score was lower, it indicated that the patients’ cardiopulmonary function and activity endurance were better [12].

The SF-36 questionnaire is the most widely used universal health questionnaire with good reliability and validity and includes 36 items, 2 fields and 8 sections. The two fields are physical and mental health. The eight sections are General Health (GH), Physical Functioning (PF), Role-Physical (RP), Bodily Pain (BP), Vitality (VT), Social Functioning (SF), Role-Emotional (RE) and Mental Health (MH). In addition to the above eight sections, the SF-36 also contains another health indicator, Health Transition (HT). The lower the score is, the greater the disability will be. The higher the score is, the lower the disability will be [13].

Excel software was used to input and collate data. Rstudio Version 1.2.1335 was used for statistical analysis. The measurement data were tested for normality. The mean ± standard deviation was used to describe normally distributed data, while the median [Q1, Q3] was used to describe non-normally distributed data. The count data were described by the frequency and composition ratio. The categorical data were tested using Chi-squared test, and non-normal measurement data were tested using the Wilcoxon rank-sum test. P < 0.05 was considered statistically significant.

## Results

No statistically significant differences were found in sex, age, preoperative pulmonary pressure, preoperative NYHA class, ejection fraction, cardiopulmonary bypass time, aortic cross-clamp time or other basic information between the groups (Table 1). Compared with the preoperative status, the PASP was significantly decreased and the LVEF was significantly increased in both groups based on the echocardiographic data at the 3-month follow up (P < 0.05). However, no significant differences were found in the PASP or LVEF between the groups at the 3-month follow up (P > 0.05). Compared with the preoperative SwanGanz catheterization data, the mPAP in the two groups was decreased significantly one year after the operation (P < 0.05). Additionally, no significant difference was found in the mPAP between the groups at the 1-year follow-up (Table 2).

**Table 2:** The comparison of the two groups from TTE and catheterization

	Group T (n = 21) (pre)	Group T (n = 21) (post)	Group C (n = 40) (pre)	Group C (n = 40) (post)	P value [1]	P value [2]	P value [3]
PASP (mmHg)	67.0 [63.0, 74.0]	53.0 [42.0, 57.0]	65.5 [62.5, 71.3]	48.5 [45.0, 56.0]	< 0.01	< 0.01	0.806
EF (%)	61.3 [54.8, 66.3]	68.2 [59.4, 70.3]	62.3 [55.4, 68.7]	68.0 [59.1, 70.3]	0.049	0.047	0.731
LVEDD (mm)	56.3 [53.8, 59.1]	54.2 [50.1, 57.3]	56.1 [49.1, 61.6]	54.1 [47.5, 58.6]	0.198	0.318	0.812
HR (times)	81.0 [75.0, 87.0]	77.0 [73.0, 83.0]	82.0 [76.0, 89.0]	80.0 [74.0, 85.3]	0.131	0.137	0.386
mPAP (mmHg)	42.0 [38.0, 47.0]	36.0 [31.0, 38.0]	43.0 [40.0, 46.3]	35.0 [32.0, 37.0]	< 0.01	< 0.01	0.985

Pre: pre-operation, Post: post-operation, ^1^Group T was compared before and after surgery, ^2^Group C was compared before and after surgery, ^3^The two groups were compared postoperatively

By comparing the results of the 6-MWD and Borg score before the operation and 3 months and 1 year after the operation between the groups of patients, the postoperative data of the two groups of patients exhibited significant improvements compared with the preoperative data (P < 0.05). Compared with the preoperative status, the median 6-MWD increased by 44 m in group T and 36 m in group C at the 3-month follow-up (P = 0.05). and increased by 40 m in group T and 38 m in group C at the 1-year follow-up (P = 0.067), with no significant difference between the groups. The median Borg score of group T decreased from 5 at baseline to 3 at the 3-month follow up and 3 at the 1-year follow up. The median Borg score of group C decreased from 5 at baseline to 4 at the 3-month follow-up and 4 at the 1-year follow-up. Statistically significant differences were found between the two groups in the Borg score at different follow-up time points, indicating that the improvement in exercise endurance and symptoms in group T was more obvious than that in group C (Tables 3, 4).

**Table 3:** The comparison of the two groups from 6-MWD in follow up

	6-MWD (Preoperative)	6-MWD (3 month)	6-MWD (1 year)	P-value [2]	P-value [3]
Group T (n = 21)	352 [312, 376]	396 [375, 443]	392 [386, 423]	0.004	0.004
Group C (n = 40)	336 [284, 375]	372 [319, 399]	374 [333, 400]	0.046	0.016
P-value^1^	0.832	0.050	0.067		

^1^Comparison of two groups of data. ^2^Comparison between preoperative and 3 month data ^3^Comparison between preoperative and 1 year data

**Table 4:** The comparison of the two groups from Borg score in follow up

	Borg score (Preoperative)	Borg score (3 month)	Borg score (1 year)	P-value [2]	P-value [3]
Group T (n = 21)	5.00[4.00, 5.00]	3.00[3.00, 4.00]	3.00[3.00, 4.00]	0.002	< 0.001
Group C (n = 40)	5.00[4.00, 5.00]	4.00[3.00, 5.00]	4.00[3.00, 4.00]	0.046	0.040
P-value^1^	0.891	0.044	0.031		

^1^Comparison of two groups of data. ^2^Comparison between preoperative and 3 month data. ^3^Comparison between preoperative and 1 year data

Comparing the data from the SF-36 questionnaire, the scores of general health, bodily pain, vitality and mental health were significantly improved in the two groups compared with those before the operation (P < 0.05). There were statistically significant differences between the two groups in general health, vitality and mental health in terms of the postoperative status, and the corresponding score in group T was better than that in group C (P < 0.05). Additionally, the score of health transition was significantly better postoperatively than preoperatively (P < 0.05) (Tables 5, 6).

**Table 5:** The comparison of the two groups from SF-36 between the preoperative and postoperative status

	Preoperation	Postoperation	P-value
*Group T (n = 21)*			
GH	30.0 [25.0, 40.0]	40.0 [35.0, 45.0]	0.007
PF	65.0 [60.0, 75.0]	75.0 [65.0, 80.0]	0.083
RP	25.0 [0.00, 50.0]	25.0 [25.0, 50.0]	0.116
BP	55.0 [45.0, 67.5]	67.5 [55.0, 77.5]	0.018
VT	35.0 [25.0, 40.0]	55.0 [40.0, 60.0]	< 0.001
SF	62.5 [50.0, 75.0]	75.0 [62.5, 75.0]	0.573
RE	66.6 [33.3, 66.7]	66.7 [66.7, 100]	0.080
MH	50.0 [44.0, 56.0]	64.0 [52.0, 68.0]	< 0.001
HT	25.0 [25.0, 25.0]	75.0 [75.0, 75.0]	< 0.001
*Group C (n = 40)*			
GH	25.0 [20.0, 35.0]	32.5 [25.0, 40.0]	0.006
PF	60.0 [53.8, 75.0]	67.5 [60.0, 76.3]	0.063
RP	25.0 [0.00, 50.0]	25.0 [18.8, 50.0]	0.236
BP	56.3 [45.0, 67.5]	67.5 [67.5, 77.5]	0.021
VT	35.0 [28.8, 41.3]	40.0 [30.0, 55.0]	0.042
SF	62.5 [50.0, 75.0]	62.5 [50.0, 75.0]	0.557
RE	33.3 [33.3, 66.7]	66.7 [33.3, 66.7]	0.204
MH	52.0 [47.0, 56.0]	56.0 [52.0, 60.0]	0.016
HT	25.0 [0.00, 31.3]	75.0 [75.0, 81.3]	< 0.001

**Table 6:** The comparison of the two groups from SF-36 in 1 year’s follow up

	Group T (n = 21)	Group C (n = 40)	P-value
GH	40.0 [35.0, 45.0]	32.5 [25.0, 40.0]	0.018
PF	75.0 [65.0, 80.0]	67.5 [60.0, 76.3]	0.102
RP	25.0 [25.0, 50.0]	25.0 [18.8, 50.0]	0.331
BP	67.5 [55.0, 77.5]	67.5 [67.5, 77.5]	0.136
VT	55.0 [40.0, 60.0]	40.0 [30.0, 55.0]	0.047
SF	75.0 [62.5, 75.0]	62.5 [50.0, 75.0]	0.218
RE	66.7 [66.7, 100]	66.7 [33.3, 66.7]	0.156
MH	64.0 [52.0, 68.0]	56.0 [52.0, 60.0]	0.014
HT	75.0 [75.0, 75.0]	75.0 [75.0, 81.3]	0.370

No significant drug-related complications, sudden deaths or rehospitalizations were observed during the follow-up period. In some patients, abdominal injection site pain occurred during subcutaneous infusion of treprostinil, but such pain was tolerable or relieved by analgesic treatment.

## Discussion

The results indicated that the application of treprostinil has a positive effect on improving the symptoms and psychological conditions of patients. Some studies had also shown that treprostinil is safe and effective in the treatment of PAH, findings that are consistent with ours [14–17]. Roela and colleagues conducted a randomized controlled double-blind trial over 24 weeks [14]. They recruited 105 patients with severe inoperable chronic thromboembolic pulmonary hypertension (CTEPH) who were subcutaneously treated with treprostinil. They found that midterm subcutaneous treprostinil is safe and effective, leading to concentration-dependent improvements in the 6-min walk distance, haemodynamics, WHO functional class, and N-terminal pro-brain natriuretic peptide levels in patients with severe non-operable CTEPH. Additionally, the application of treprostinil in children has received increasing attention. Marilyne and colleagues undertook a four-centre retrospective cohort study [15]. They reviewed all available clinical, echocardiographic and haemodynamic data on children with PAH who were treated with a continuous subcutaneous infusion of treprostinil between 2009 and 2016. They concluded that subcutaneous treprostinil infusion is an effective therapy without serious side effects in children with PAH.

Valvuloplasty and valve replacement are effective methods to treat valvular heart disease. However, in China, most patients are seriously ill when they visit the hospital, and most patients can only have valvular replacement to treat valvular diseases [18]. A large amount of clinical and literature data has shown that, after left heart valve replacement, the PAH of some patients could be significantly reduced after a short period, while that of other patients was not significantly changed [19, 20]. Early diagnosis and treatment are necessary for patients with left heart valvular disease complicated with PAH [21]. Presently, few studies exist on the effect and safety of treprostinil in the treatment of PAH associated with left heart valve disease. We reported our preliminary experience with early postoperative administration of treprostinil in these patients, and our results showed that treprostinil could improve the early postoperative prognosis of these patients [22]. This study was continued to explore the midterm effect of treprostinil on postoperative discharge of theses patients who undergo mechanical valve replacement.

This single-centre retrospective study demonstrated that surgery could effectively improve the PAH and LVEF of most patients based on TTE and catheterization. However, the difference between groups T and C in the above data was not statistically significant. Because the number of cases in our study was small, we suspected that a study with a larger sample might yield more positive findings. Additionally, the 6-MWD and Borg score were compared between the groups of patients: the median 6-MWD was increased and the Borg score was lower in both groups, and the Borg score in the group T was lower than that in group C at the 3-month and 1-year follow up. Although the difference between the groups was not statistically significant in 6-MWD at the 3-month and 1-year follow up, considering that the p-value was close to 0.05, we believed that, if the sample size was larger, the difference might be statistically significant.

In addition to solving patients’ anatomical deformities, attention should also be given to patients’ mental health and social functions. The SF-36 questionnaire is a quality-of-life rating questionnaire widely used in clinical practice that mainly focuses on patients’ subjective feelings towards their physiology and psychology. After analysing the data from the SF-36, the scores of patients in some sections of physiological and psychological fields were better than the preoperative scores. The cause might be that the patient’s illness was cured after artificial valve replacement, making them feel better physically and mentally. The improvements in GH, VT and MH were better in group T than in group C. The results indicated that the application of treprostinil has a positive effect on improving the symptoms and psychological conditions of patients.

In the literature on subcutaneous injection of treprostinil, the most common adverse reaction was pain at the injection site. The related literature showed that common painkillers or pain relief patches could be used to relieve pain, a finding that was consistent with ours [23]. However, some studies also proposed that subcutaneous infusion of treprostinil would produce reactions related to prostacyclin, such as diarrhoea and jaw pain, which were not observed in our study [21].

The present study possessed some limitations. First, because the subjects were patients at one hospital, mainly permanent residents in local provinces, the representativeness might be poor. Second, the treprostinil administration method was only subcutaneous continuous infusion. Currently, oral and inhaled administration methods are available worldwide, and the prognosis and safety of other administration methods for such patients need to be further studied. Third, subjective assessment might be due to the placebo effect, as the patients on the new drug had a device for administration and the study was not blinded, that should be recognized as an important limitation. This study was a retrospective analysis. There may be some selection bias, but the study supports our conclusion to some extent. Next, we hope to carry out a prospective, randomized, controlled study and a larger sample size study to further verify the conclusion.

## Conclusion

Continuous subcutaneous infusion of treprostinil was not capable of decreasing pulmonary pressures in patients with severe left heart valvular disease combined with moderate or severe PAH during 1 year follow-up, although which some of our data suggest that might improve the symptoms and quality of life of these patients.

## Data Availability

The datasets generated and/or analysed during the current study are not publicly available due that we assured patients and their families that the data would only be used for academic research and their privacy would be protected when we signed the informed consent, but are available from the corresponding author on reasonable request.

## Abbreviations

PAH: Pulmonary hypertension
6-MWT: 6-Min walk test
TTE: Transthoracic echocardiography
PASP: Pulmonary artery systolic pressure
LVEDD: Left ventricular end-diastolic dimension
HR: Heart rate
mPAP: Mean pulmonary artery pressure
MWD: 6-Min walking distance
GH: General health
PF: Physical functioning
RP: Role-physical
BP: Bodily pain
VT: Vitality
SF: Social functioning
RE: Role-emotional
MH: Mental health
HT: Health transition
CTEPH: Chronic thromboembolic pulmonary hypertension
